# A *de novo* transcriptome assembly approach elucidates the dynamics of ovarian maturation in the swordfish (*Xiphias gladius*)

**DOI:** 10.1038/s41598-019-43872-6

**Published:** 2019-05-14

**Authors:** Giorgia Gioacchini, Luca Marisaldi, Danilo Basili, Michela Candelma, Paolo Pignalosa, Riccardo Aiese Cigliano, Walter Sanseverino, Gary Hardiman, Oliana Carnevali

**Affiliations:** 1 Department of Life and Environmental Sciences (DISVA), Marche Polytechnic University (UNIVPM), 60131 Ancona, Italy; 2 OCEANIS srl, 80056 Ercolano (NA), Italy; 3 Sequentia Biotech, 08193 Bellatera (BCN), Spain; 4 School of Biological Sciences & Institute for Global Food Security, Queens University Belfast, BT9 5AG Belfast, UK

**Keywords:** Ichthyology, Transcriptomics

## Abstract

The Mediterranean swordfish (*Xiphias gladius*) has been recently classified as overfished and in 2016, the International Commission for the Conservation of the Atlantic Tunas (ICCAT) established a multi-annual management plan to recover this stock. To successfully achieve this goal, knowledge about swordfish biology is needed. To date, few studies on swordfish have been performed and none of them has provided useful insights into the reproductive biology at molecular level. Here we set to characterise the molecular dynamics underlying ovarian maturation by employing a *de novo* transcriptome assembly approach. Differential gene expression analysis in mature and immature ovaries identified a number of differentially expressed genes associated with biological processes driving ovarian maturation. Focusing on ovarian steroidogenesis and vitellogenin uptake, we depict the molecular dynamics characterizing these processes while a phylogenetic analysis let us identify a candidate vitellogenin receptor. This is the first swordfish transcriptome assembly and these findings provide in-depth understanding of molecular processes describing ovarian maturation. Moreover, the establishment of a publicly available database containing information on the swordfish transcriptome aims to boost research on this species with the long-term of developing more comprehensive and successful stock management plans.

## Introduction

The swordfish (*Xiphias gladius* Linnaeus, 1758) is a large, solitary, fast-swimming and highly migratory species with a worldwide distribution. The unique phenotype of this species, in addition to its peculiar heat and lubricating organs^1,2^, is thought to contribute to its exceptional predatory behaviour and swimming capacities, which make the swordfish one of the fastest swimmers in the pelagic realm. Owing to the fact that the swordfish is a species of strong commercial interest, valuable fisheries were established from the late 1950 s. However, since that time, robust management plans have been lacking due to the scarcity of information on reproduction, growth, sexual maturity and migratory behaviour. Such a lack of knowledge is also caused by clear logistic constraints associated with collecting samples in pelagic areas which often require collaboration with fishermen and *ad-hoc* sampling surveys. According to a recent stock assessment report by the International Commission for the Conservation of the Atlantic Tunas (ICCAT)^3^, the Mediterranean swordfish stock was classified as “overfished and currently suffering overfishing” and a recovery plan was subsequently established including measures such as Total Allowable Catches (TAC), fishing fleet capacity limitations, closed fishing season and a minimum size^4^. Nonetheless, the current size of minimum catch of 100 cm (Lower Jaw to Fork Length, LJFL) set is far below the 140 cm (LJFL) size of first maturity (L50) for the Mediterranean swordfish as found by De la Serna *et al*.^5^. Previous studies provided insights into swordfish reproductive biology in the north-western Atlantic^6^ and in the Mediterranean area^7,8^, however, they do not provide comprehensive details on gonadal development and puberty onset necessary to determine the reproductive potential and to assess the status of the stock. Accordingly, deeper knowledge on swordfish gonad development is required to establish the duration of the spawning season, spawning pattern and reproductive dynamics as well as to deliver scientific data to policy makers in order to improve current stock management models.

In fish, puberty occurs following gonadal sex differentiation and is characterized by the capacity of fish to produce for the first time in its life, mature gametes^9^. It is well known that the hypothalamus-pituitary-gonadal (HPG) axis plays a key role in regulating puberty in vertebrates^10^. Gonadotropin releasing hormone (GnRH) from the hypothalamus stimulates the synthesis and release of the gonadotropins follicle stimulating hormone (FSH) and luteinizing hormone (LH) from the anterior pituitary, which in turn act on the gonads inducing oogenesis and spermatogenesis mainly through the stimulation of gonadal steroidogenesis. Although this cascade has been well characterized in many fish species^11,12^, the mechanisms underlying the onset of puberty are not fully understood. Since gonadal development is not entirely modulated by upstream signals, it might be postulated that key factors involved in puberty onset should be investigated at the gonad level possibly acting via autocrine/paracrine mechanisms. Here, we generated a high-quality transcriptome assembly using an RNA-sequencing approach in order to investigate local molecular dynamics driving puberty onset in the swordfish. As a major focus of this study, a pathway-based analysis was employed to specifically depict the ovarian steroidogenesis and vitellogenin synthesis and uptake molecular networks. In addition, by exploring the phylogenetic relationship of the Low-Density Lipoprotein Receptor (LDLR) superfamily a candidate vitellogenin receptor was identified. This work represents the first swordfish *de novo* transcriptome assembly and such findings provide the additional knowledge needed to refine current ICCAT recovery plan towards a successful conservation of the Mediterranean swordfish.

## Results

### Transcriptome assembly

RNA sequencing of samples on Illumina HiSeq2500 platform generated 606.560.486 million of raw reads of which 531.938.284 million were maintained after trimming and low-quality filtering steps (see Supplementary Table S1). The raw assembly produced 185.901 transcripts ranging from 201 to 18.293 nt with an N50 value of 2.492 nt and an average size of 1.325 nt while the average GC content was 46%. Further cleaning of raw transcripts resulted in a final assembly of 100.869 transcripts with an N50 value of 2.037 nt, an average size of 937 nt and an average GC content of 44% (see Supplementary Table S2). Furthermore, 83.13% of the reads were successfully mapped back to the assembled transcriptome of which 96% mapped uniquely while 99% and 98.2% matched sets of single copy eukaryotic and Metazoa genes, respectively. The percentage of duplicated and fragmented transcripts accounted for 9.5%.

### Transcripts annotation

The SwissProt, TrEMBL, GO and KEGG databases were employed for annotation of the 100,869 sequences. A total of 31,704 (31.4%) sequences were successfully associated to a gene name. More specifically, 3,584 (3.5%) and 28,120 (27.9%) sequences matched the SwissProt and the TrEMBL databases, respectively. These database queries found *X. gladius* sequences to closely match sequences of *Fundulus heteroclitus* (20.2%), *Oreochromis niloticus* (13%), *Larimychthys crocea* (11%), teleosts (10.7%) and *Danio rerio* (5.7%). In the final transcriptome assembly 30,398 (30.1%) sequences had a significant match against the GO database, of which 37.7% representing biological processes, 27% associated with cellular components and 35.2% matching molecular functions. Sequences matching the KEGG database accounted for up to 18,158 (18%).

### Drivers of ovary maturation

**Figure 1:**
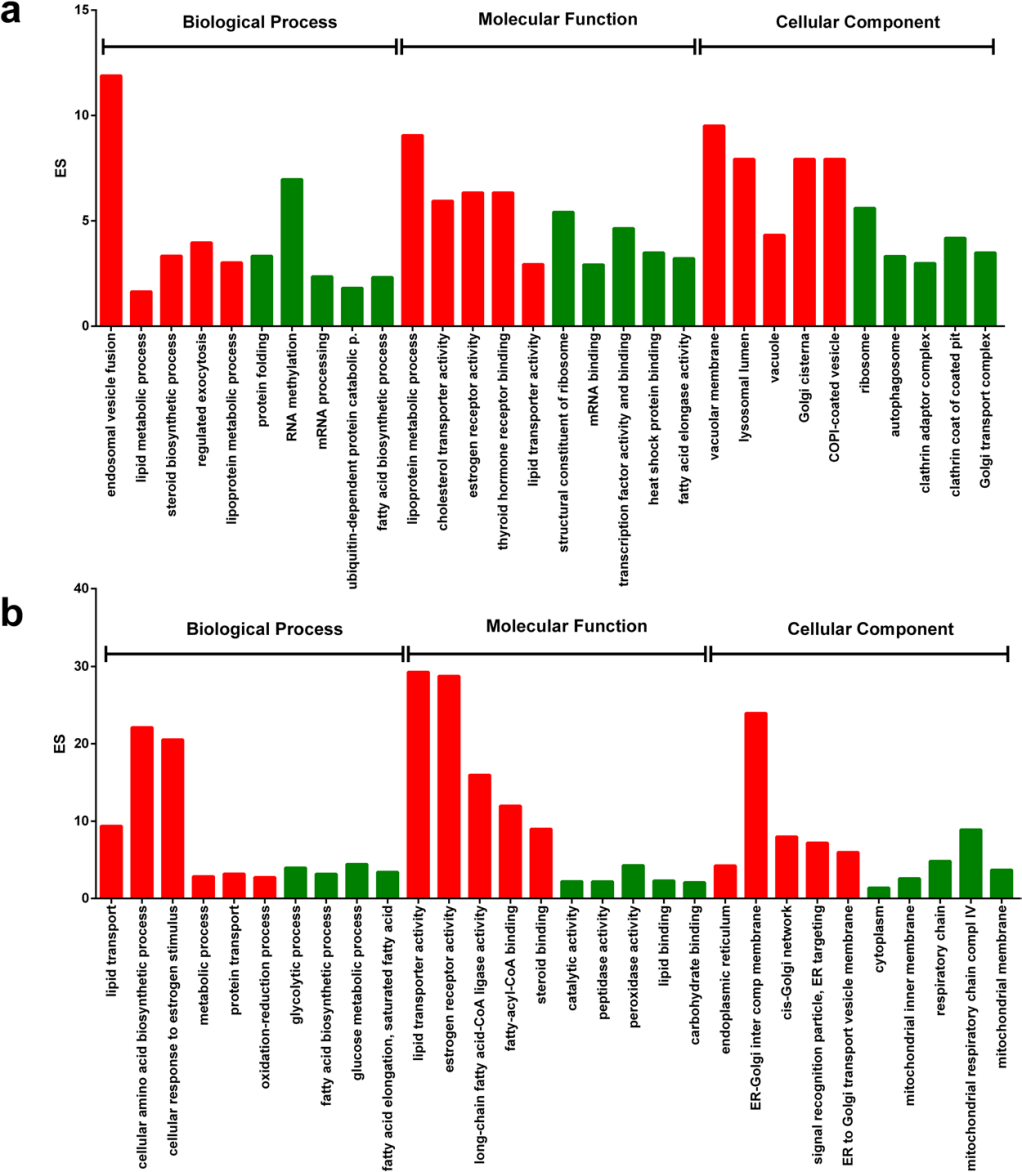
Gene Ontology differences underlying immature and mature ovaries (**a**) and livers. (**b**) The bar plots show the Gene Ontology terms enriched of genes differentially expressed between mature and immature ovaries and livers. The y-axis represents the enrichment score (ES) and gene ontology terms have been organized according to the three main categories of Biological Process, Molecular Functions and Cellular Components. Red and green bars stand for terms up-regulated and down-regulated in the mature tissues compared with the immature ones, respectively.

Since the overarching goal of our study was to investigate the molecular dynamics underlying ovarian maturation, we focused our effort in the identification of differentially expressed genes between mature and immature ovaries and livers. Before running DEA, a PCA was employed to assess the reproducibility and general quality of the analysis performed (see Supplementary Fig. S1). The overall variance explained by the first two principal components accounted for up to 97.8% and the first principal component best discriminated mature and immature ovaries with 92.5% of explained variance. DEA identified 6,501 transcripts, of which 4,211 functionally annotated, to be differentially expressed between immature and mature ovaries at 1% FDR. Gene Ontology Biological Processes (BP) known to drive and regulate ovarian maturation such as steroid biosynthetic process (GO:0006694), endosomal vesicle fusion (GO:0034058), lipoprotein metabolism (GO:0042157) and cholesterol transporter activity (GO:0017127) were significantly up-regulated while mRNA processing (GO:0006397), protein folding (GO:0006457), ribosome (GO:0005840), fatty acid biosynthetic process (GO:0006633) and clathrin adaptor complex (GO:0030131) were down regulated in mature ovaries (Fig. 1a). These results showed consistent agreement with the KEGG Pathways Analysis, in which ribosome biogenesis (ko03008), RNA transport (ko03013), mRNA surveillance (ko03015), oocyte meiosis (ko04114), cell cycle (ko04110) and fatty acid elongation (ko00062) were significantly down-regulated while endocytosis (ko04144) and cholesterol metabolism (ko04979) were up regulated in mature ovaries (see Supplementary Tables S3, S4). Interestingly, ovarian steroidogenesis, a key pathway involved in the progression of ovarian maturation, was found to be enriched when considering genes differentially expressed at 5% FDR.

A total of 2,914 transcripts, of which 1,834 functionally annotated, were found to be differentially expressed between immature and mature livers at 1% FDR. Gene Ontology Biological Processes (BP) known to characterize a mature liver such as Lipid transport (GO:0006869), Estrogen receptor activity (GO:0030284), steroid binding (GO:0005496), metabolic process (GO:0008152) and endoplasmic reticulum (GO:0005783) were significantly up-regulated while glycolytic process (GO:0006096) and fatty acid biosynthetic process (GO:0006633) were down regulated in mature livers (Fig. 1b). These findings were in agreement with the KEGG Pathways Analysis as estrogen signalling pathway (Ko04915) and protein processing in endoplasmic reticulum (ko04141) were significantly up-regulated while glycolysis/gluconeogenesis (ko00010) and pyruvate metabolism (ko00620) were down regulated in mature livers (see Supplementary Tables S5, S6).

**Figure 2:**
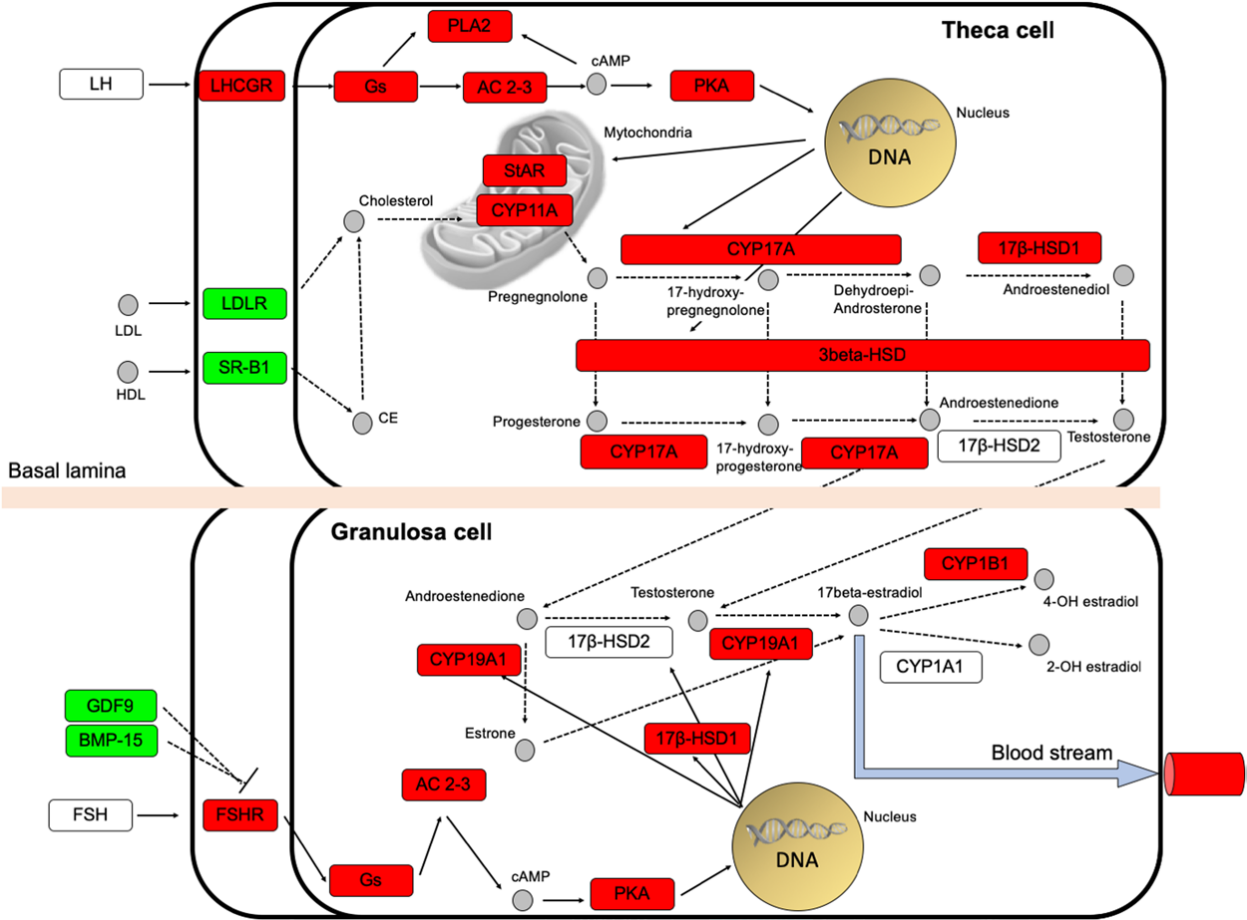
Molecular network of ovarian steroidogenesis adapted from KEGG. Genes up-regulated and down-regulated in the mature ovaries are shown in red and green, respectively, while genes not identified as differentially expressed are displayed in white. Solid lines represent direct interaction while Dotted lines stand for indirect interaction.

**Figure 3:**
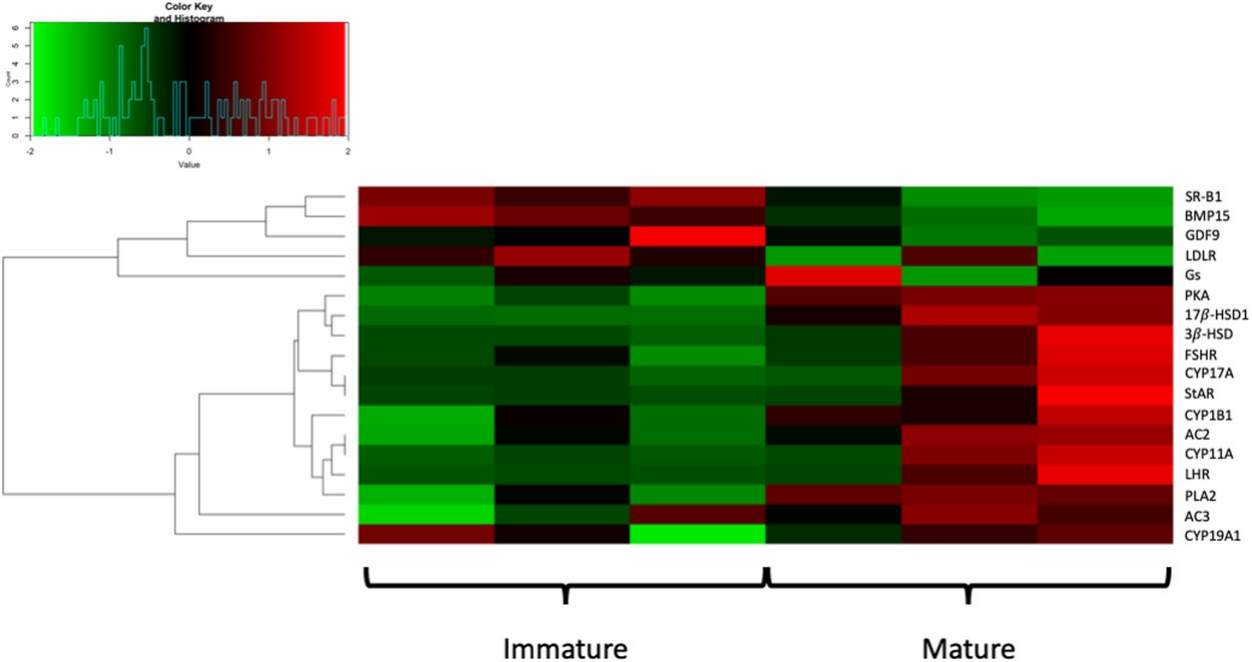
Heatmap of genes involved in the ovarian steroidogenesis pathway. The heatmap shows the behaviour of genes involved in the ovarian steroidogenesis pathway. Red and green are assigned to high and low values of expression, respectively, according to the reference plot.

**Figure 4:**
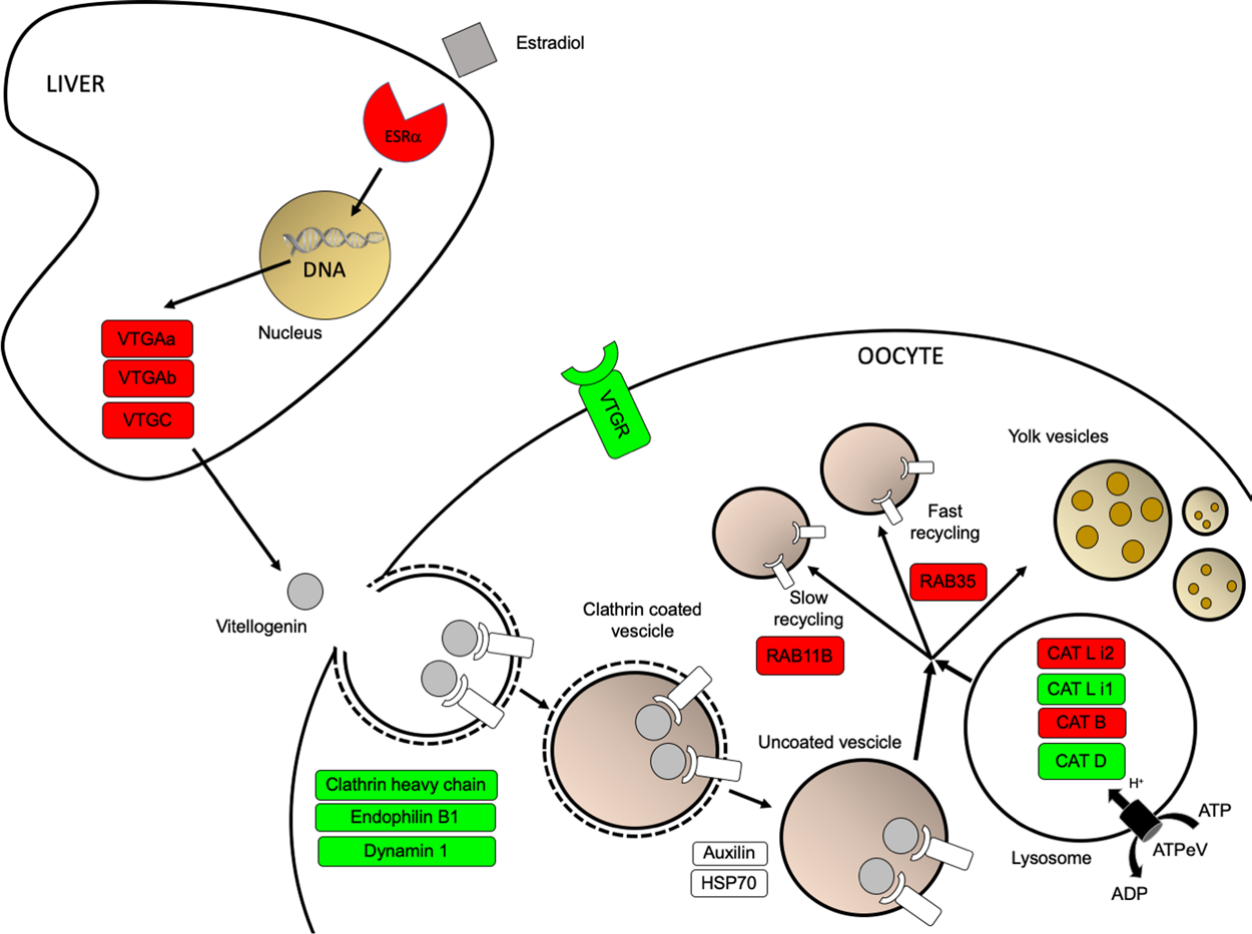
Molecular network of vitellogenin synthesis and uptake. Genes up-regulated and down-regulated are shown in red and green, respectively, while genes not identified as differentially expressed are displayed in white.

**Figure 5:**
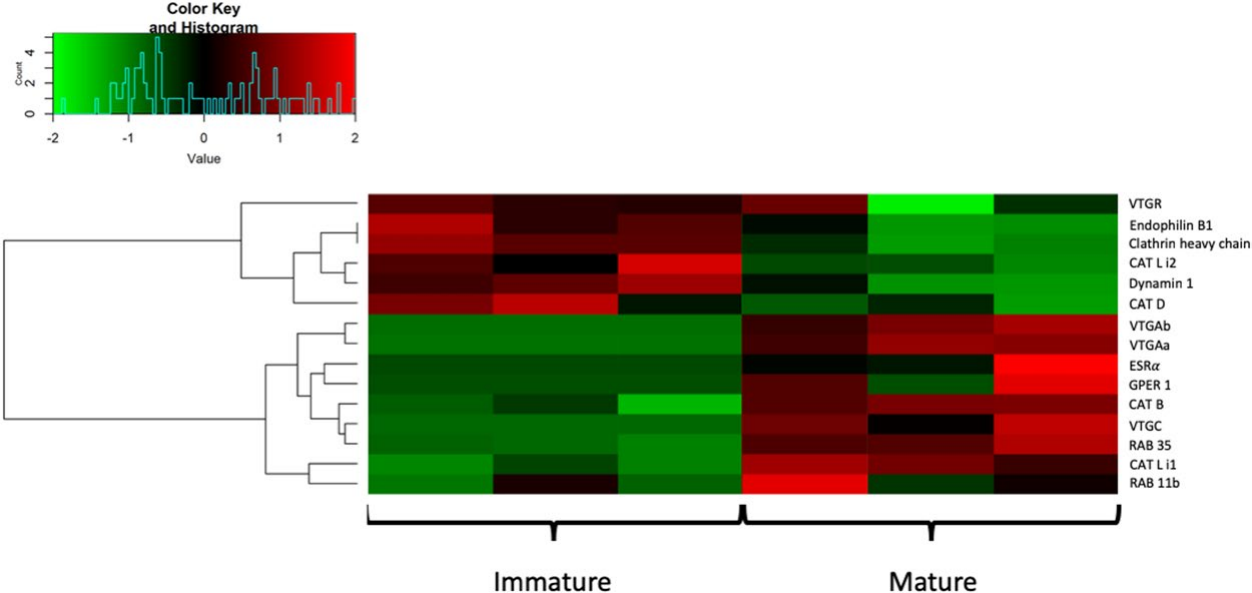
Heatmap of genes known to be involved in both the synthesis and uptake of vitellogenin. Red and green are assigned to high and low values of expression, respectively, according to the reference plot.

A major focus on the steroid biosynthesis pathway revealed that the entire enzymatic cascade producing key sex steroids (i.e. E2, 17a,20b-DP, 11-KT) as well as the *fsh* and *lh* receptors were up-regulated in mature ovaries (Figs 2, 3). Interestingly, the Low-Density Lipoprotein Receptor (*ldlr*) and the Scavenger Receptor Class B member 1 (*srb1*) displayed down regulation during vitellogenesis. Moreover, the Bone Morphogenetic Protein 15 (*bmp15*) and the Growth and Differentiation Factor 9 (*gdf9*) were found to be down-regulated in mature ovaries. A representation of the vitellogenin (*vtg*) synthesis and uptake pathway in the swordfish ovary was then established (Fig. 4) and represented along with the gene’s expression profile (Fig. 5).

In the liver, the organ where vitellogenin is mainly synthesised, we identified the estrogen receptor α (*esr*α) to be up-regulated in livers of mature individuals. When investigating vitellogenin, three different forms, *vtgAa, vtgAb* and *vtgC*, were identified to be up-regulated in livers of mature individuals. Based on experimental approaches aimed at the identification of vtg receptors in other teleost species in combination with the analysis of LDLR phylogenetic relationship in the present work, we identified a candidate vtg receptor likely to play a role in the vitellogenin uptake which exhibited higher expression in pre-vitellogenic ovaries. Accordingly, clathrin coated pits related transcripts such as clathrin heavy chain, endophilin B1 and dynamin also displayed higher expression in pre-vitellogenic ovaries. Moreover, lysosomal cathepsins D and L isoform 1 were downregulated while cathepsins B and L isoform 2 were upregulated in mature ovaries. Key Rab proteins *Rab11a*, involved into the slow endocytic recycling towards cell surface, and *Rab35*, involved into the fast recycling, were upregulated in vitellogenic ovaries.

**Figure 6:**
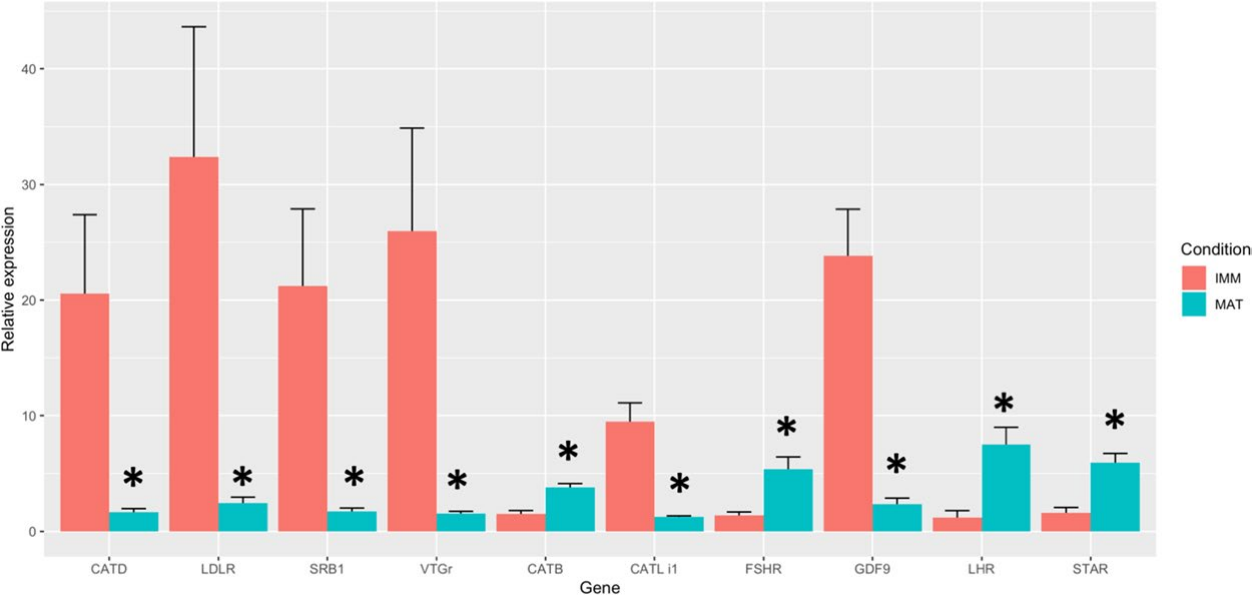
qPCR validation. Bar plot shows gene expression levels of key genes, measured by qPCR, involved in either the ovarian steroidogenesis or the vitellogenin uptake pathways. Significance between mature and immature ovaries for each gene is shown by an asterisk and standard error reported with error bars.

Gene expression profiles of a few genes were not always consistent between replicates due to the swordfish being an asynchronous spawner where oocytes at different developmental stages are found. In addition, some degree of variability was expected being the swordfish a wild species. Some of the key genes identified in the aforementioned pathways were quantified by qPCR and differences were validated (Fig. 6).

### Low-density lipoprotein receptor superfamily

**Figure 7:**
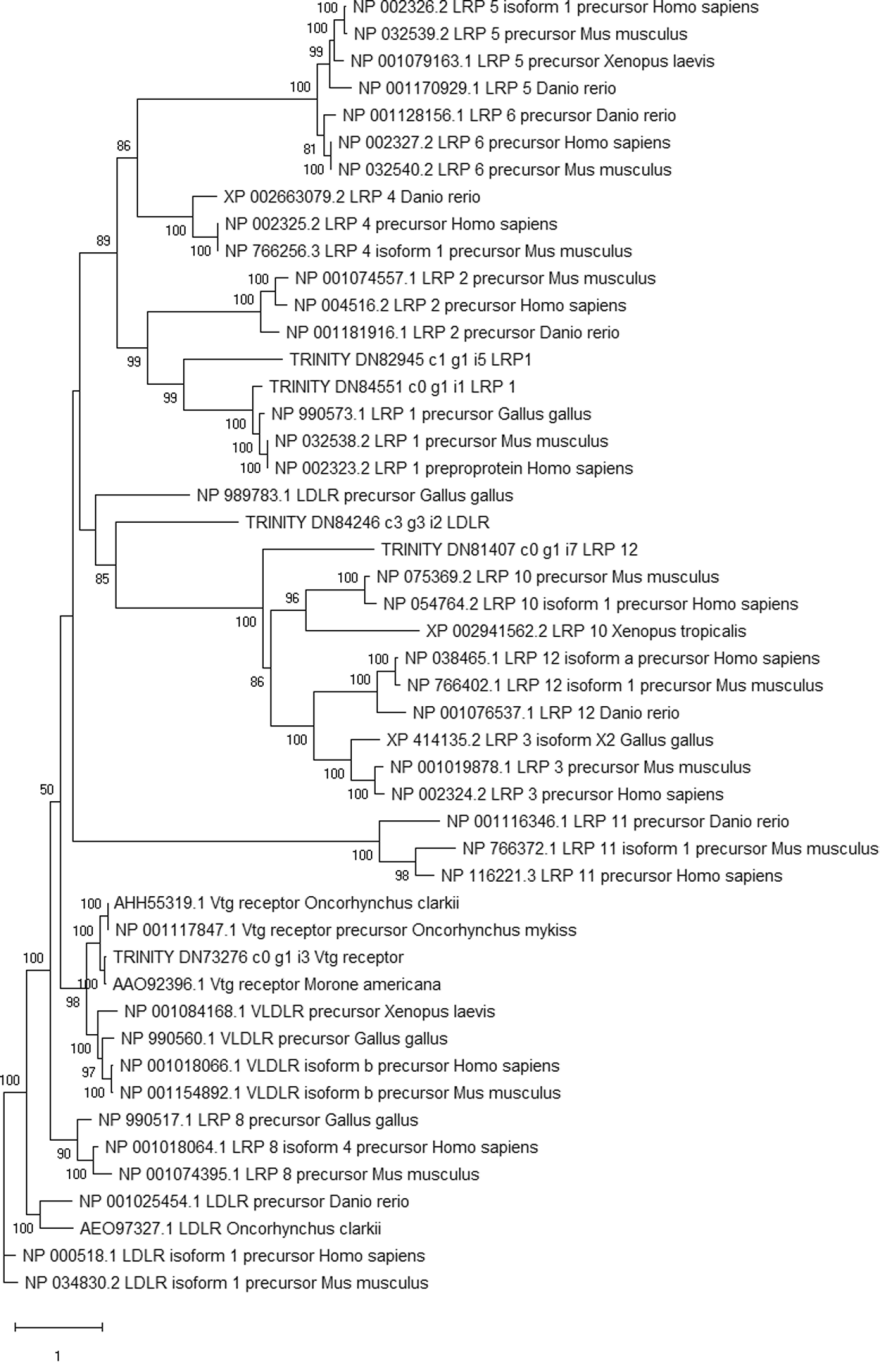
Phylogenetic tree of low-density lipoprotein receptors. The phylogenetic tree shows relationships, based on sequence similarity, between low-density lipoprotein receptors found in swordfish (labelled as TRINITY probes) and in other species. Bootstrap values are shown at each node.

The phylogenetic tree showing relationship between LDLR orthologs species displayed conserved clusters for each receptor type belonging to this superfamily (Fig. 7). Two well separated clusters supported by high bootstrap values were made up by two spliced variants VLDLR^+^ and VLDLR^−^, the latter being the vitellogenin receptor in teleost fishes. Taking advantage of this phylogenetic approach, we were successfully able to identify a potential vitellogenin receptor candidate in the swordfish clustering with VLDLR^−^ sequences and the amino acid sequence included a signal peptide region, eight low-density lipoprotein receptor domain class A (LDLa), a calcium-binding epidermal growth factor-like domain (EGF_CA), four low-density lipoprotein-receptor YWTD domain (LY) and a transmembrane domain. However, in the swordfish transcriptome sequences matching the *lrp13*, a novel receptor which has been recently suggested as an additional vitellogenin receptor in a few teleost species, were absent.

## Discussion

A wealth of studies applying High-Throughput technology has steadily increased in recent years with enhanced insights into reproduction, growth, metabolism and toxicological responses in non-model species in the wild^13–16^. To serve as a resource for future studies of swordfish biology, we have successfully sequenced, assembled and annotated the transcriptome of the swordfish *Xiphias gladius*, starting from several tissues such as female gonads, liver, intestine and stomach. As first extent, we specifically focused on data obtained from mature and immature ovaries in order to improve our understanding of specific molecular pathways driving sexual maturation in this species and to enhance both conservation and management of this resource for the Mediterranean fisheries.

Teleost sexual maturation is triggered by a complex interplay between environmental stimuli (i.e. photoperiod and temperature), energy balance, social interactions, physiological state and reproduction strategy^17^. Female maturation relies on the production of viable eggs achieved by the loading of maternal molecular cargo in oocytes which underlies the survival and proper development of embryos. Such maternal investment mainly consists of VTG-derived yolk proteins, polar (i.e. phosphoglycerides) and neutral (i.e. triacylglycerol) lipids, vitamins and minerals^18^. Internalization of this bulk is one of the main outputs of the HPG reproductive endocrine axis that chemically links brain centres, able to convey environmental information, with the ovary through systemic signals in addition to autocrine/paracrine factors operating at the gonadal level. Indeed, the general overview provided by the Gene Ontology and KEGG pathways approaches showed up-regulation in mature ovaries of processes related to steroid biosynthesis, lipoprotein metabolism and vesicle trafficking. Nevertheless, such a molecular bulk is not restricted only to nutrients and structural components but also include a plethora of maternal transcripts with key functions during early stages of developing embryos. Accordingly, occurrence of RNA methylation as well as mRNA maturation processes were found to be higher in immature ovaries supporting their pivotal role in early stage of ovary development. Intriguingly, RNA methylation is a recently discovered process and is involved into direct regulation of protein expression, RNA stability and mRNA translation^19^, thus it is likely to play a significant role in the molecular tuning needed throughout the oogenesis.

It is well known that E2 synthesis in follicular cells with its subsequent release in the blood stream is the main response upon circulating FSH stimulation and leads to hepatic vitellogenin synthesis^20,21^. Nevertheless, at the ovary level, synthesis of E2 and 17,20 DP was respectively linked also to oogonial proliferation and initiation of the first meiotic division in the common carp *Cyprinus carpio* and Japanese huchen *Hucho perryi*^22^. In the present study, the two gonadotropin receptors *lhr* and *fshr* were up-regulated in mature ovaries, in which vitellogenic oocytes were present, and so did the entire downstream cascade of enzymes (i.e. *20β-HSD*, *17α-hydroxylase, aromatase P450*, *StaR*) leading to sex steroids synthesis in the follicular layer. On the other hand, the receptors *ldlr* and *srb1*, thought to be involved into the internalization of LDL and HDL and thus representing the starting point for cholesterol uptake and steroid biosynthesis^23,24^, were down-regulated in vitellogenic ovaries, suggesting an accumulation of transcripts during early stages of oogenesis prior to active nutrient uptake with subsequent translation and recycling of the protein products during lipidic and vitellogenic stages as found in the cutthroat trout *Oncorhynchus clarkii*^25^. Increasing levels of *lhr* transcripts in mature ovaries might also be indicative of vitellogenesis completion of a certain batch of oocytes and its subsequent entry into final maturational stages. Indeed, the final maturation of fully-grown follicles is unambiguously driven by a surge of systemic LH which leads to completion of meiosis, switch of the steroidogenic pathway from E2 to progestin synthesis, oocyte progestin responsiveness and finally hydration^11^. However, oocytes and follicular cells are not just passively regulated by endocrine signals but they play an active role to orchestrate follicle development through autocrine/paracrine signals such as modulation of gonadotropin signals by means of locally produced factors belonging to the transforming growth factor beta (TGF-β) superfamily^26,27^. In addition to their well-known role in gametogenesis and follicle development, these factors regulate steroidogenesis and final oocyte maturation via paracrine mechanisms from mammals to fish^28,29^. Indeed, the oocyte-derived *bmp15* was able to regulate ovarian steroidogenesis by suppressing *fshr* expression in rat granulosa cells^30,31^. Direct *in vitro* evidences of such BMP-mediated paracrine modulation in teleost was found in the zebrafish *Danio rerio*, in which both *bmp2b* and *bmp4* suppressed *fshr* expression and up-regulated *lhr* in co-cultured oocytes and follicle cells^32^. Moreover, in the European sea bass *Dicentrachus labrax*, a reciprocal and positively correlated pattern of gene expression was found between *bmp15* and *gdf9*, with highest expression in pre-vitellogenic oocytes followed by a sharp decline at the onset of vitellogenesis, suggesting a transcriptional control of gonadotropin receptor expression by BMP15 and GDF9 and/or vice versa^33^. Consistent with such a view, human chorionic gonadotropin (hCG), known to act through the LHR in zebrafish, was able to down-regulate *gdf9* expression in fully-grown oocytes *in vitro*^34^. Here, *bmp15* and *gdf9* were down-regulated in vitellogenic ovaries, highlighting the importance of the local autocrine/paracrine control of follicle development and, due to their differential expression, suggesting their possible involvement into regulation of steroid biosynthesis and follicle gonadotropin responsiveness in the swordfish.

Vitellogenin is synthesized in the liver under E2 control, released into the blood and endocyted by growing vitellogenic oocytes in the ovary by means of specific receptors^35^. Multiple vitellogenin forms are found in teleost species due to teleost specific whole genome duplication which dates back about 320 million years ago^36^. A direct consequence of such duplication is the evolution of a complex ligand-receptor system composed by several vtg receptors with specific ligand binding affinity^37–39^ and by yet to discover mechanisms of regulation which lead to a different ratio of plasmatic and endocytosed VTG forms into the oocyte^40,41^. Here, three forms of vitellogenins were identified and found to be up-regulated in the livers of sexually mature females. This would fit well with the evolutionary history of this molecule since three functional forms are found in other fish species^36,42^. It is clear that hepatic vitellogenin synthesis is up-regulated by estrogens and that the main receptor involved into this response is the *esr*α^43^. According to these previous findings, the swordfish *esr*α was up-regulated in the livers of sexually mature females caught during the reproductive season, supporting the role of *esr*α as the main estrogen receptor isoform involved into E2-induced vitellogenin production in the liver. One of the aims of the present study was the identification and characterization of the receptor involved in such uptake by analysing the pattern of expression and exploring phylogenetic relationship. The candidate vtg receptor (VLDLR^−^) exhibited higher expression in pre-vitellogenic ovaries, a trend observed also in the cutthroat trout *Oncorhynchus clarkii* and *Morone* species^38,44^. Such expression would be related to an intense transcription prior to vitellogenesis followed by both synthesis and recycling of the protein with subsequent depletion of the mRNA pool. Accordingly, the molecular machinery of clathrin-coated pits was down-regulated in mature ovaries, consistent with the idea of pre-vitellogenic transcription and both slow and fast recycling of protein products throughout vitellogenenesis^45^. The phylogenetic tree showed well conserved clusters of LDLR family members across vertebrates even if from distantly related species. The candidate vitellogenin receptor formed a cluster with respective orthologues and such cluster was closely related to *vldlr*^+^. The close relationship between *vldlr*^−^ and *vldlr*^+^ was investigated with multiple approaches which revealed the two receptor as spliced variants, the former lacking the O-linked sugar domain and directly linked to vitellogenin uptake^38^. The deduced structure of swordfish vtg receptor showed typical LDLR family domains with high homology to vtg receptors of many species^46–48^. Functional roles of the core receptors architecture include LDLa consecutive domains thought to interact with the receptor-binding domains of the vitellogenin^49^ and the six-bladed β-propeller structure made up by EGF domains and YWTD repeats which mediates the pH dependent ligand release^50^. Interestingly, there were no sequences significantly matching the *lrp13*, a novel vitellogenin receptor recently found in a few teleost species^39,44^. Nonetheless, different reproductive strategies, ecological traits and phylogenetic lineages characterizing teleosts might reflect multiple selective models of vitellogenin uptake, processing and accumulation. For instance, it has been shown that the ligand-binding domain of the vitellogenin receptor interacts also with Apoliprotein B/E in the *Oreochromis aureus* suggesting a coevolution of the two systems and indicating off-target binding of such receptors^51^. Establishing comprehensive principles of how multiple vitellogenin forms (VtgAa, VtgAb, VtgC) are taken up by growing oocytes across species still remains a challenge and major differences likely exists in terms of ligand specificity and subsequent accumulation of derived protein products^37^. Overall, the question if other receptors, other than *vldlr*^*−*^, are responsible for VTG internalization in the swordfish still remain to be answered and will require further investigation at the protein level. Once VTG is internalized into growing oocytes it is cleaved by cathepsins into yolk protein components (YP) (lipovitellins, phosvitin, ß’-component and C-terminal component). A well-established pathway in fish oocytes begins with the cathepsin D, responsible for the first proteolytic cleavage of vitellogenin, then a second step occurs with cathepsins L or B depending on the species^52–54^. Indeed, species spawning pelagophil and benthophil eggs undergo high and low level of final hydration, respectively. Water uptake in pelagic eggs is mainly driven by a proteolytic process occurring at around the time of germinal vesicle breakdown which generates a pool of free amino acids. Intriguingly, a teleost-specific aquaprorin (Aqp1ab) mediating water uptake during the final maturation processes has been recently characterized at genomic, phylogenetic and molecular level in marine species and in some freshwater species spawning partially hydrated eggs^55^. Such pronounced difference in eggs water content between pelagophil and benthophil species underlies divergent proteolytic processing of the YPs as well as species-specific ecological traits and habitat usage. In the zebrafish *Danio rerio*, a freshwater species spawning benthic eggs, cathepsin B activates cathepsin L since cathepsin L activity was suppressed by a cathepsin B inhibitor^56^. Important differences between species exist even though eggs with similar water content are spawned. Indeed, Fabra and Cerdà found that in the killifish *Fundulus heteroclitus*, a benthophil species, the mRNA levels of cathepsin L transiently accumulated in maturing oocytes while those of cathepsin B remained stable throughout the whole oogenesis^57^. Nonetheless, cathepsin B enzyme activity was higher during oocyte maturation and it is considered the major protease involved into YPs hydrolysis in this species^58^. On the other hand, in the sea bream *Sparus aurata*, a pelagophil species, the expression of cathepsins B, D, L decreased from early vitellogenic oocytes through hydrated eggs but the enzyme activity well correlated with transcript levels only for cathepsin B^59^. Interestingly, the coho salmon *Oncorhynchus kisutch* showed respectively decreasing and stable mRNA levels of cathepsin B and D in oocytes entering the cortical alveoli stage^60^. However, expression of cathepsin D and L increased in oocytes acquiring maturational competence in another salmonid species, the rainbow trout *Oncorhynchus mykiss*^61^. In the swordfish, we found the cathepsin D, B and two cathepsin L isoforms to be differentially expressed between vitellogenic and pre-vitellogenic ovaries. Previously, three different cathepsin L isoforms were identified in the genome of zebrafish with different pattern of expression in adult tissues and during embryogenesis^62^. In the swordfish transcriptome, cathepsin L isoform 1, whose expression decreased in vitellogenic ovaries, is likely to play a role in YPs processing since exhibited nearly 20 to 25-fold higher expression than the other isoform. Furthermore, swordfish cathepsin D and B exhibited down-regulation and up-regulation in vitellogenic ovaries, respectively. According to the findings of Carnevali and co-workers, in the sea bream, the pattern of expression of cathepsin D showed decreasing mRNA levels through the vitellogenesis^59^ while cathepsin B displayed an opposite trend. These result might underlie several features typical of such enzymes across teleost species: (i) cathepsins are first synthetized as zymogen, pre-pro enzymes which are then activated by a drop in lysosomal pH^63,64^ and this would reflect the scarce correlation found between mRNA levels and enzyme activities; (ii) different expression patterns were found also in species spawning eggs with similar characteristics in term of final water content. To sum up, it is hard to clearly establish an overall pattern of cathepsin expression across species during the oogenesis since such differences likely underlie different reproductive strategies and ecological traits.

In conclusion, according to the ICCAT plan, recovery of the Mediterranean swordfish stock has at least a 60% probability of success. Despite this percentage represents an optimistic goal, filling the gap of knowledge about swordfish biology of reproduction has the potential to significantly improve the predicted scenario. In this context, application of highly sensitive molecular technologies with the ability to generate “big omics data” offers the potential to drive new understanding of molecular functions. More specifically, RNA sequencing approaches provide a powerful tool to investigate the molecular mechanisms of key biological processes. In this context, we employed the power of *de novo* transcriptome assembly to shed light on the swordfish molecular dynamics occurring when vitellogenesis starts. We successfully depicted the ovarian steroidogenesis and vitellogenin uptake molecular pathways and identified a potential candidate for vitellogenin receptor in this species. Although biological processes driving reproduction are well conserved, especially between fish, differences in molecules driving a given biological pathway may arise. As a consequence, further effort investigating isoforms could potentially increase our understanding of the swordfish reproductive biology. Moreover, we set up a dedicated database (www.swordfishomics.com) that is publicly available where all the information about the swordfish transcriptome can be directly accessed by the scientific community. This offers a powerful tool and the integration of our findings with other approaches as stock assessment methodologies has the potential to refine the current ICCAT recovery plan for the conservation of the Mediterranean swordfish.

## Materials and Methods

### Sample collection and experimental design

Swordfish were captured in July 2017 in the central Mediterranean Sea (38°22′66″N; 12°27′30″E) by longliners. Ovary, stomach, intestine, and liver samples were collected from a total of 10 individuals, placed in RNAlater (Ambion, Austin, TX, USA), stored at 4 °C for 16 h and then transferred to −20 °C until RNA extraction. Small ovary pieces (2 cm^3^) were fixed in formaldehyde-glutaraldehyde and stored at 4 °C until histological analyses in order to confirm the reproductive status of each specimen. The fish were caught for commercial purpose and ovaries samples were collected according to the International Commission for the Conservation of Atlantic Tuna (ICCAT) guidelines for biological sampling. The procedures did not include animal experimentation, and ethics approval is not necessary in accordance with the Italian legislation (D.L. 4 of Mars 2014, n. 26, art. 2).

### Histological assay

Ovaries were processed as described in Forner-Piquer *et al*.^65^. Briefly, after fixation in formaldehyde-glutaraldehyde, samples were dehydrated in a series of alcohol baths, cleared in Xylene and finally embedded in paraffin. Sections 5 µm thick were cut with a microtome and stained with Mayer’s haematoxylin – eosin. The histological slides were observed under a Zeiss Aixio Imager M2 microscope and microphotographed with a high-resolution camera Zeiss Axiocam 105 color. Female samples were classified as mature or immature depending on the most abundant oocyte developmental stage (see Supplementary Fig. S2). Immature female size and gutted weight were 111 ± 3.4 cm (LJFL) and 14.8 ± 2.1 Kg, respectively, while size and gutted weight of mature female were 154.6 ± 9.6 cm (LJFL) and 49.2 ± 6.3 Kg, respectively.

### RNA isolation, library preparation and sequencing

Total RNA was isolated from a total of 17 tissue samples from 10 animals: 3 ovaries from immature females and 3 ovaries from mature females, 3 livers from immature females and 3 livers from mature females, 3 livers from mature males, 1 stomach and 1 intestine both from mature males. Isolation of total RNA was carried out by RNeasy Plus Mini Kit (Qiagen, Dusseldorf, Germany) according to the manufacturer’s instructions. The RNA integrity and concentration were determined by Nanodrop-2000 spectrophotometer (Thermo Fisher Scientific, USA) and Agilent 2100 Bioanalyzer (Agilent, Santa Clara, CA). Genomic DNA was removed by applying RNase-free DNase I (Qiagen) after a treatment of 30 min at 37 °C. Libraries were created with the Illumina TruSeq Stranded mRNA Library Prep Kit and then sequenced with an HiSeq2500.

### *De novo* transcriptome assembly and annotation

Illumina paired-end 150 bp reads from 17 *X* *. gladius* samples were processed to produce the transcriptome assembly. Raw reads were trimmed and clipped with BBDuk^66^ setting a minimum Phred-like quality of 35 and a minimum length of 35 nucleotides. The quality of the reads before and after trimming was checked with the software FASTQC^67^. Possible contaminant reads were removed using the GAIA metagenomics suite (www.metagenomics.cloud) using a database of bacteria, archaea, virus, protists and fungi as reference. High quality reads were then normalized with Trinity^68^ using the following options:–SS_lib_type RF–pairs_together–max_cov 50. *De novo* transcriptome assembly was then performed with Trinity using the options:–SS_lib_type RF–no_normalize_reads–min_kmer_cov 2–KMER_SIZE 32–min_per_id_same_path 95. The longest isoform for each gene was extracted with Trinity and then redundancy was removed with CD-HIT-EST^69^ using the following options: -r 0 -g 1. Kallisto^70^ was used to calculate transcripts expression using the normalized reads and only the transcripts with more than 1 TPM (transcripts per million reads) were retained. Transrate^71^ was used to extract the quality statistics about the assembly, whereas the BUSCO (v3)^72^ pipeline was used to check the presence of Eukaryotic and Metazoan conserved genes. *In silico* transcriptome translation was performed with TransDecoder.LongOrfs^73^, the obtained peptides were blasted (BLASTp, minimum evalue 0.00001) against the NCBI NR (downloaded on January 2017) and the UniProt databases (downloaded on January 2017) and the results were given to TransDecoder.Predict to obtain the final dataset of predicted proteins.

Functional annotation of the transcriptome was performed following the AHRD pipeline^74^. First, the predicted proteins were queried (BLASTp, evalue 0.00001) against the UniProt database of Metazoa and the TrEMBL database of Osteoglossocephalai (both downloaded on January 2017). The resulting files were used as input for the AHRD script including the Gene Ontology annotation of the UniProt database. KEGG annotation was performed using the KEGG Automatic Annotation Service (KaaS)^75^.

### Transcriptome expression quantification and differential expression analysis

Transcript expression quantification was performed using Kallisto with the Trinity final assembly and the reads from the 17 samples as input. The raw estimated counts were normalized with two approaches: TPM (Transcripts Per Million) and TMM (Trimmed Mean Normalization of M-values). The TPM were used for plotting purposes. The TMM normalization was used specifically for the differential expression analysis. To assess the reproducibility of the technical replicates and to look for potential outliers a PCA analysis was performed on the TMM normalized counts using the “prcomp” function within the R statistical environment. Differential expression analysis between mature and immature ovaries and livers was carried out within the R statistical environment using the NOISeq package^76^. For the differential expression analysis (DEA), lowly expressed transcripts were removed and transcripts with a probability of being differentially expressed higher than 0.99 (corresponding to FDR < = 1%) were kept. A Gene Ontology Enrichment Analysis (GOEA) on the differentially expressed transcripts was performed with an in-house script based on the method described in^77^ and applying a 1% FDR threshold. To facilitate the biological interpretation, a KEGG enrichment analysis to identify the most enriched pathways across the up- and down-regulated genes was performed using the “phyper” function within the R statistical environment (1% FDR). Pathways enriched for genes found to be differentially expressed between both the GIF and the GMF and the LIF and LMF groups were then further investigated using the search&color pathway KEGG web tool^78^. Given the objective of our study and in order to perform a more comprehensive analysis, the ovarian steroidogenesis and vitellogenin synthesis and uptake pathways were investigated considering genes differentially expressed at 5% FDR. Our rationale was that the FDR threshold must be considered in the context and releasing the stringency to 5% was to guide perform systems level analyses and avoid false negatives, where crucial data is lost. Heatmaps of differentially expressed genes involved in the selected pathways were drawn employing the “heatmap.2” function within the R statistical environment and using “spearman” and “average” as distance measure and agglomeration method, respectively.

### Teleost low-density lipoprotein receptor (LDLR) orthologs

Following Mushirobira and co-workers^39^, multiple sequences belonging to the LDLR superfamily from several species were downloaded from NCBI. Moreover, from the swordfish transcriptome, transcripts annotated as LDLR which displayed full-length Open Reading Frame (ORF) were selected and conserved domains as well as structural features were assessed by SMART^79^ and InterProScan^80^. This strategy allowed us to identify 5 full-length sequences from the swordfish transcriptome and 44 orthologue sequences in other species. Deduced amino acids sequences were first aligned with Muscle^81^ and then the least informative portions of the alignment were trimmed with TrimAl^82^. Finally, a maximum likelihood tree was created with the IQ-Tree suite^83^ applying 1000 bootstrap replicates as phylogeny test and WAG as substitution model^84^.

### Experimental validation

Validation of key genes involved into steroidogenesis (*fshr*, *lhr*, *srb1* and *StaR*) and vitellogenin synthesis and uptake (cathepsin B, D, L i1 and the vtg receptor) was performed by means of qPCR. Briefly, from samples selected for transcriptomics analysis, a total amount of 1 µg of RNA was used for cDNA synthesis, employing the iScript cDNA Synthesis Kit (Bio-Rad). PCRs were performed with the SYBR green method in a CFX96 Real-Time PCR system (Bio-Rad) following Gioacchini and co-workers^85^. Four replicates per condition were used. Acidic ribosomal phosphoprotein P0 (*arp*) and Ribosomal protein L7 (*rpl7*) were used as internal standards in order to standardize the results by eliminating variation in mRNA and cDNA quantity and quality. No amplification products were observed in negative controls and no primer-dimer formations were observed in the control templates as indicated by the melting curve analysis. The data obtained were analysed using the CFX Manager Software version 3.1 (Bio-Rad), including GeneEx Macro Conversion and GenEx Macro files and results represented by bar-plots along with the standard error. Statistical significance was attained using a t-test. Specific primer pairs for target genes (see Supplementary Table S7) were designed with Primer-Blast^86^.

### Database

The web server uses NodeJS (version 8.9.4) together with the Express framework (version 4.16) in the back-end. Requested data is saved into files, which are read by the back-end and provided to the front-end, which mainly consists of HTML/CSS and JavaScript, in the corresponding format (e.g. tables or images). The site also contains a BLAST section, in which the user can query any sequence against the transcriptome with blastn (version 2.2.30+)^87^. A freely available JBrowse (version 1.12.3)^88^ was also integrated to the site, thus allowing visual inspection of the new transcriptome assembly, the UniProt/TrEMBL matches against the predicted proteins, and the RNA-seq expression.

## Supplementary information

Supplementary information

## Data Availability

Raw sequencing data was deposited as FASTQ files in NCBI Sequence Read Archive (SRA) database under the Bioproject number PRJNA515417. Statistics of the assembly, sequence annotation and results of differential expression analysis are publicly available at www.swordfishomics.com.
