# Supplementary material for: A de novo transcriptome assembly approach elucidates the dynamics of ovarian maturation in the swordfish (Xiphias gladius)
Source: Sci Rep. 2019 May 14;9:7375. doi: 10.1038/s41598-019-43872-6 (PMC6517582; doi:10.1038/s41598-019-43872-6)
Supplement: Supplementary file 1 — Supplementary information [file 41598_2019_43872_MOESM1_ESM.pdf]

Supplementary Information

____

A *de novo* transcriptome assembly approach elucidates the dynamics of ovarian maturation in the swordfish (*Xiphias gladius*)

Authors

Giorgia Gioacchini[1a], Luca Marisaldi[1a], Danilo Basili[1a], Michela Candelma[1], Paolo Pignalosa[2], Riccardo Aiese Cigliano[3], Walter Sanseverino[3], Gary Hardiman[4], Oliana Carnevali[1]

Affiliation

[1]Department of Life and Environmental Sciences (DISVA), Marche Polytechnic University (UNIVPM), 60131 Ancona, Italy
[2]OCEANIS srl, 80056 Ercolano (NA), Italy
[3]Sequentia Biotech, 08193 Bellatera (BCN), Spain
[4] School of Biological Sciences & Institute for Global Food Security, Queens University Belfast, BT9 5AG Belfast, UK

Corresponding author

Oliana Carnevali (o.carnevali@univpm.it)

[a] These authors contributed equally to this work

Sample ID	Assigned ID	Raw reads	Cleaned reads
**Gonad immature female 1**	GIF1	41.202.936	36.483.926
**Gonad immature female 2**	GIF2	40.329.320	35.169.144
**Gonad immature female 3**	GIF3	35.525.804	32.050.208
**Gonad mature female 1**	GMF1	34.854.888	30.193.696
**Gonad mature female 2**	GMF2	37.081.988	31.760.626
**Gonad mature female 3**	GMF3	44.615.566	38.000.088
**Intestine**	INT	26.925.730	24.346.536
**Liver immature female 1**	LIF1	33.142.636	28.967.198
**Liver immature female 2**	LIF2	38.512.492	33.346.540
**Liver immature female 3**	LIF3	37.934.870	33.160.278
**Liver mature female 1**	LMF1	35.053.400	31.313.634
**Liver mature female 2**	LMF2	40.364.134	35.523.746
**Liver mature female 3**	LMF3	32.117.194	28.265.148
**Liver mature male 1**	LMM1	31.870.620	27.800.940
**Liver mature male 2**	LMM2	35.993.946	31.486.178
**Liver mature male 3**	LMM3	30.007.468	25.816.452
**Stomach**	STO	31.021.494	28.253.746

Table S1

Statistic	Value
**n sequences**	100.869
**Smallest (bp)**	201
**Longest (bp)**	18.293
**n bases (Mbp)**	95.52
**Mean length (bp)**	937.15
**N50**	2.037
**GC%**	44

Table S2

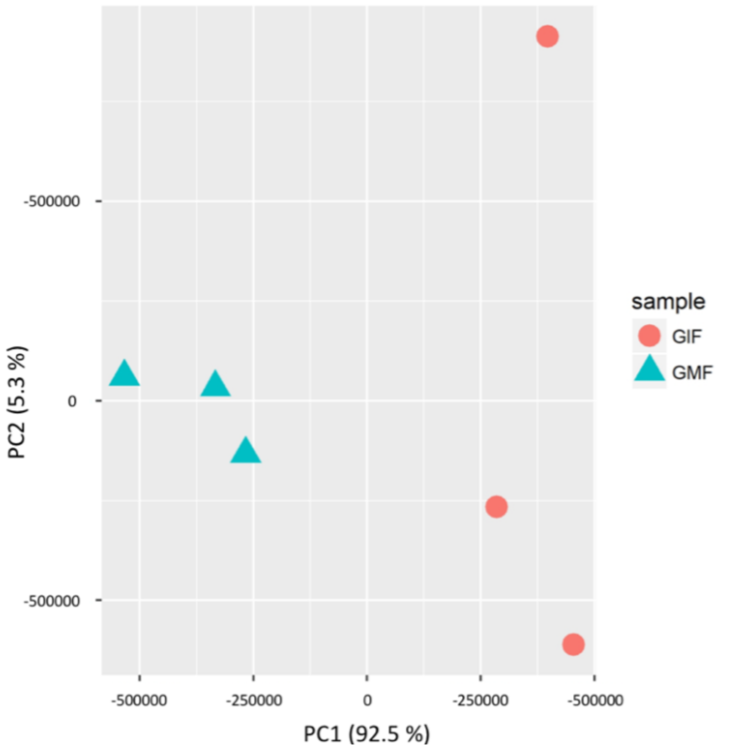

Fig. S1

KEGG Pathway	FDR	ES	N° genes
Cholesterol metabolism	$8.89x10^{-7}$	3.3	18
Phosphatidylinositol signaling system	0.0002	2.6	16
Glycerolipid metabolism	0.0003	3.1	9
PI3K-Akt signaling pathway	0.0005	1.7	45
Inositol phosphate metabolism	0.001	2.5	14
PPAR signaling pathway	0.002	2.4	14
Axon guidance	0.002	1.9	23
Fc gamma R-mediated phagocytosis	0.003	2.2	15
AMPK signaling pathway	0.003	2	20
HIF-1 signalling pathway	0.007	2.1	14
Insulin signaling pathway	0.007	1.9	18
Endocytosis	0.009	1.6	37
Longevity regulating pathway	0.009	2	17

Table S3

KEGG pathway	FDR	ES	N° genes
**Ribosome**	$2.6x10^{-21}$	3.2	62
**Splicesome**	$1.3x10^{-20}$	3.2	52
**RNA transport**	$1.7x10^{-12}$	2.5	55
**Ribosome biogenesis in eukariotes**	$4.4x10^{-7}$	2.5	30
**Necroptosis**	$2.7x10^{-4}$	2	20
**mRNA surveillance pathway**	$3.1x10^{-4}$	2.2	21
**RNA degradation**	$3.1x10^{-4}$	2.2	22
**Cell cycle**	$7.2x10^{-4}$	1.9	29
**Phagosome**	0.003	1.9	20
**Fatty acid elongation**	0.008	2.9	8
**Proteasome**	0.008	2.1	16
**Oocyte meiosis**	0.008	1.8	19

Table S4

KEGG Pathway	FDR	ES	N° genes
**Fatty acid degradation**	$2.39x10^{-8}$	12.3	8
**PPAR signaling pathway**	$1.19x10^{-5}$	7	7
**Protein processing in endoplasmic reticulum**	$1.93x10^{-5}$	4.1	13
**Glycerophospholipid metabolism**	$1.76x10^{-3}$	4.7	8
**Estrogen signaling pathway**	$4.12x10^{-3}$	4.1	6

Table S5

KEGG Pathway	FDR	ES	N° genes
**Metabolism of xenobiotics by cytochrome P450**	0	8.5	5
**Complement and coagulation cascades**	$4.0x10^{-27}$	6.3	31
**Pentose and glucuronate interconversions**	$3.41x10^{-9}$	6.8	7
**Glycolysis / Gluconeogenesis**	$2.50x10^{-8}$	4.5	15
**Cysteine and methionine metabolism**	$2.19x10^{-7}$	4.4	13
**Insulin signaling pathway**	$2.19x10^{-7}$	3	20
**Retinol metabolism**	$6.36x10^{-7}$	5.1	7
**Adipocytokine signaling pathway**	$7.12x10^{-7}$	3.6	15
**Steroid hormone biosynthesis**	$9.87x10^{-6}$	4.6	7
**Leukocyte transendothelial migration**	$1.19x10^{-6}$	3	18
**Proximal tubule bicarbonate reclamation**	$1.54x10^{-6}$	5.9	7
**HIF-1 signaling pathway**	$2.33x10^{-6}$	3.1	18
**Tyrosine metabolism**	$4.51x10^{-6}$	4.7	8
**Glucagon signaling pathway**	$8.09x10^{-6}$	3.1	15
**Drug metabolism - other enzymes**	$9.86x10^{-6}$	4.7	5
**Tight junction**	$1.56x10^{-5}$	2.4	21
**AMPK signaling pathway**	$2.01x10^{-5}$	2.7	18
**Carbohydrate digestion and absorption**	$2.06x10^{-5}$	4.8	7
**Glyoxylate and dicarboxylate metabolism**	$2.52x10^{-5}$	4.1	11
**Starch and sucrose metabolism**	$3.12x10^{-5}$	4.3	8
**Mineral absorption**	$3.96x10^{-5}$	3.9	10
**Glycerolipid metabolism**	$4.31x10^{-5}$	3.7	9
**Pyruvate metabolism**	$6.08x10^{-5}$	3.8	10
**Glutathione metabolism**	$6.08x10^{-5}$	3.8	8
**Methane metabolism**	$7.85x10^{-5}$	4.6	7
**One carbon pool by folate**	$7.84x10^{-5}$	4.6	7

Table S6

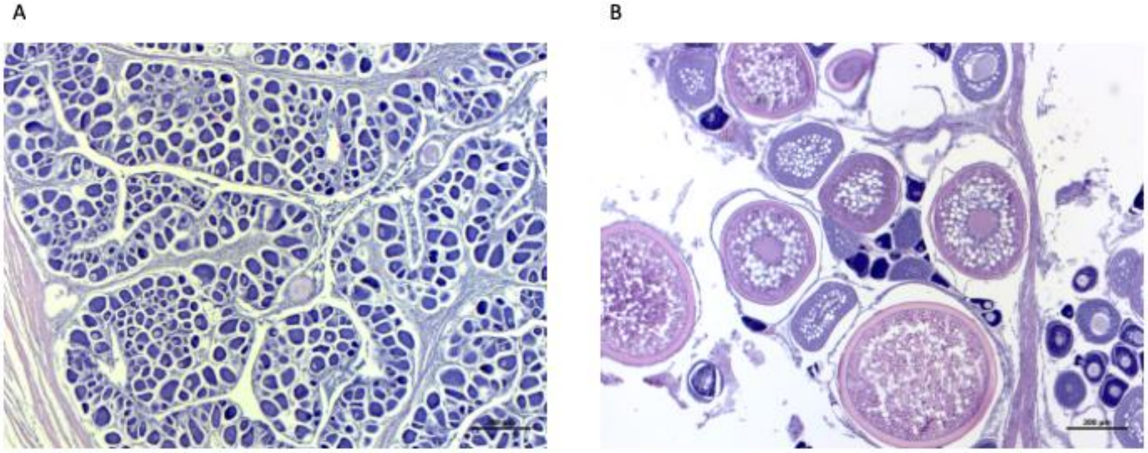

Fig. S2

Gene name	Transcript ID	Fw primer 5’ to 3’	Rv primer 5’ to 3’	Product length (bp)	Tm (°C)
**catb**	TRINITY_DN76699_c2_g1_i4	GGACTGGTCTCTGGAGGTCT	ATGCACTTCGGTGTGTCTCC	131	60°C
**catd**	TRINITY_DN82105_c4_g1_i4	CGAAGAGCACTGGCGCA	CAGGTCGTAGAAGGAGCAGTG	191	60°C
**catl**	TRINITY_DN81187_c2_g2_i6	ACGGCTACAAGCACAAAACG	ACGGCTACAAGCACAAAACG	123	60°C
**fshr**	TRINITY_DN78062_c0_g1_i3	TCCAAACTGACCGTTCCTCG	GCCGCAGAGCATGTGTAATG	256	60°C
**lhr**	TRINITY_DN84789_c1_g2_i1	TGTGTAACCTGGCTTTCGCT	GCAGCCTCTTGTTGACATGC	243	60°C
**srb1**	TRINITY_DN83748_c0_g2_i1	GGCCATCAGTAGATCCAAAGTG	AGAAAGCACCGTCCCGAAAA	79	60°C
**star**	TRINITY_DN75922_c0_g1_i2	GGCAAACGGAGACAAAGTCC	ATTAGGGTTCCACTCCCCCA	145	60°C
**vtgr**	TRINITY_DN73276_c0_g1_i3	TAAGGAGCCCTGCCTGATCT	TGGGCACCATCATCTCTTCG	207	60°C
**ldlr**	TRINITY_DN84554_c1_g2_i11	TACCTGCGTCTGTCCCGATA	GCTGTGCTCCTGAGTGTGTT	195	60°C
**gdf9**	TRINITY_DN69915_c0_g1_i3	AAAGCCCCACTGGAGTTCAC	GCTCTGGTTTTGGCACCATC	163	60°C
**rpl7**	TRINITY_DN75764_c1_g2_i3	GTACTGCTCGCAAAGTGGGA	GACTTTGGGGCTGACACCAT	98	60°C
**arp**	TRINITY_DN75205_c4_g2_i1	ACAGCCCAGTCTTTCCACAG	TTTAAGGTCCGGGCAACCTG	74	60°C

Table S7

FIGURES AND TABLES LEGEND

Table S1: **Raw reads statistics**. The table provide information about both the number of raw reads generated by the sequencing platform and those obtained after the filtering steps for each sample.

Table S2: **Transcriptome assembly statistics**. The table provide the final transcriptome assembly statistics as the transcriptome total number of sequences, transcript mean length as well as the length of both the longest and the shortest transcript, the n bases, the N50 value and percentage of CG content.

Fig. S1: **Mature and immature ovary relationships**. The Principal Component Analysis (PCA) shows relationships between samples. A nice separation between immature and mature ovaries is detectable on the PC1 (92.5% of the total variance). Despite one of the immature samples is away from the other replicates on the PC2, it only accounts for the 5.3% of the variance. GIF and GMF stands for immature gonad and mature gonad, respectively.

Table S3: **Kegg pathways over-expressed in mature ovaries**. The table shows the list of Kegg pathways found to be enriched of genes up-regulated in the mature ovaries with respect to the immature ones at 1% FDR. FDR, enrichment score and number of genes found to be differentially expressed within each pathway are reported.

Table S4: **Kegg pathways over-expressed in immature ovaries**. The table shows the list of Kegg pathways found to be enriched of genes down-regulated in the mature ovaries with respect to the immature ones at 1% FDR. FDR, enrichment score and number of genes found to be differentially expressed within each pathway are reported.

Table S5: **Kegg pathways over-expressed in mature livers**. The table shows the list of Kegg pathways found to be enriched of genes up-regulated in the mature livers with respect to the immature ones at 1% FDR. FDR, enrichment score and number of genes found to be differentially expressed within each pathway are reported.

Table S6: **Kegg pathways over-expressed in immature livers**. The table shows the list of Kegg pathways found to be enriched of genes down-regulated in the mature livers with respect to the immature ones at 1% FDR. FDR, enrichment score and number of genes found to be differentially expressed within each pathway are reported.

Fig. S2: **Ovaries histological analysis**. Samples were classified as immature (A) or mature (B) depending on the most advanced oocyte developmental stage. The immature ovary is almost entirely represented by oocytes at the perinucleolar stage while in the mature ovary oocyte at the vitellogenin stage are the most abundant.

Table S7: **qPCR primers**. The table provide the list of primers, along with their trinity transcript id, forward and reverse sequences, product length and annealing temperature employed for the experimental validation by qPCR of key genes involved in either the ovarian steroidogenesis or vitellogenin uptake pathways.
